# Sleep loss suicidal ideation: the role of trait extraversion

**DOI:** 10.3389/fnbeh.2022.886836

**Published:** 2022-10-20

**Authors:** William D. S. Killgore, Michael A. Grandner, Andrew S. Tubbs, Fabian-Xosé Fernandez, Tracy Jill Doty, Vincent F. Capaldi II, Natalie S. Dailey

**Affiliations:** ^1^Department of Psychiatry, University of Arizona College of Medicine, Tucson, AZ, United States; ^2^Walter Reed Army Institute of Research, Silver Spring, MD, United States; ^3^Department of Psychology, University of Arizona, Tucson, AZ, United States

**Keywords:** sleep, suicide ideation, depression, personality, extraversion, individual differences

## Abstract

**Background:** It is known that sleep disturbance is associated with increased suicidal thinking. Moreover, completed suicides, when adjusted for the proportion of the populace that is awake at a given time, are more probable during the late night/early morning hours. Despite these concerns, no studies have examined the role of trait-like individual differences in vulnerability to suicidal ideation during sleep deprivation or insomnia. In two separate studies, we examined whether the trait of extraversion is predictive of changes in suicidal thinking following two nights of sleep deprivation and among individuals meeting the criteria for insomnia.

**Methods:** Study 1: Twenty-five healthy military personnel (20 males), ages 20–35 completed the NEO-PI-R Extraversion scale and the Suicidal Ideation (SUI) scale of the Personality Assessment Inventory (PAI). Participants completed 77 h of continuous sleep deprivation. After 56 h of sleep deprivation, participants completed the SUI scale a second time. We predicted a change in SUI scores from baseline extraversion. Study 2: 2,061 adults aged 18–79 (900 males) were divided into two groups based on the clinical threshold (≥ 10) on the Insomnia Severity Index (ISI) and completed measures of extraversion and depression, including the suicide item of the Patient Health Questionnaire-9 (PHQ9).

**Results:** Study 1: After controlling for the caffeine group and changes in PAI Depression, Extraversion scores were used to predict changes in SUI scores using stepwise multiple linear regression. Higher Extraversion was significantly associated with increased non-clinical suicidal ideation following sleep loss, β = 0.463, partial *r* = 0.512, *p* = 0.013. Study 2: After controlling for depression, the effect of insomnia on suicidal ideation was moderated by trait extraversion (*p* < 0.0001). Overall, the presence or absence of insomnia had little effect on individuals low in trait extraversion (i.e., introverts), but insomnia was associated with significantly higher suicidal ideation among high trait extraverted individuals.

**Conclusions:** Higher trait extraversion was associated with increased vulnerability to suicidal ideation between rested baseline and total sleep deprivation and was associated with greater suicidal ideation among those meeting criteria for clinically severe insomnia. These findings point to a potential trait-like vulnerability factor that may further our understanding of sleep disruption in the phenomenology of suicide.

## Introduction

Suicide is currently the 10th leading cause of death in the United States and its incidence steadily increased between 1999 and 2018, with a slight decline in 2019, according to the Centers for Disease Control and Prevention (CDC, 2022). Overall, by 2019, there were 13.9 suicides per 100,000 people, and suicide is now the second leading cause of death among adolescents and younger adults under the age of 35 (CDC, 2022). Suicide has also been a particularly concerning issue for the U.S. military, as the rates have increased from 20.3 suicides per 100,000 active-duty Service members in 2015 to 28.7 per 100,000 as of data available in 2020 (DoD Annual Suicide report). A recent report concluded that since 9/11, military suicides have claimed the lives of more than four times as many U.S. Service members as the number who were killed over the same period as a result of combat operations (Suitt, 2021). While suicide is a major public health issue, the absolute number of individuals who die by completed suicides generally remains around 1.4% around the globe (Bachmann, 2018). Despite the severity of the problem, it has been remarkably difficult to predict completed suicides on an individual level, as the associated factors leading to a decision to commit suicide are complex and multifactorial. Suicidal ideation—thinking about suicide as an option—is relatively common among depressed individuals, but far fewer will actually carry out a suicide attempt. Similarly, suicide attempts are about 30 times more common than completed suicides (Bachmann, 2018). While it is true that most individuals who complete suicide have shown evidence of major depressive disorder or other emotional struggles (Coryell and Young, 2005), most people with depression do not complete or even attempt suicide (Brådvik, 2018). Thus, completed suicide is difficult to predict and is associated with many complex and disparate factors.

It is becoming increasingly apparent that sleep problems and circadian disruption may play a particularly important role in suicidal thinking and behavior. Sleep disorders, including insomnia, parasomnias, and apnea have each been independently associated with increased suicidal tendencies (Pigeon et al., 2012; Bishop et al., 2018, 2020; Britton et al., 2019), and there is growing evidence that being awake at times that are out of sync with one’s circadian phase may increase the risk for suicidal ideation and behavior (Perlis et al., 2016a, b; Tubbs et al., 2020). Lack of sleep and being awake at the wrong times both appear to influence neurobehavioral systems that sustain normal emotional homeostasis. Sleep loss has been shown to reduce positive mood state (Grezes et al., 2021; Stenson et al., 2021), degrade emotional intelligence (Killgore et al., 2008, 2021), increase feelings of persecution (Kahn-Greene et al., 2007), and reduce frustration tolerance (Kahn-Greene et al., 2006), all of which can influence an individual’s outlook. Without sufficient restorative sleep, people make poorer decisions and take more risks under certain conditions (Killgore et al., 2006; Dickinson et al., 2021), particularly when relying on emotional valuation cues (Whitney et al., 2015). Mechanistically, many of these changes appear to be due to alterations in functional activity and neural communication throughout the brain produced by sleep deprivation (Venkatraman et al., 2007). In particular, neuroimaging findings show that sleep loss weakens the functional connectivity between the regulatory systems of the ventromedial prefrontal cortex and the emotional activating systems of the amygdala, leading to reduced ability to modulate emotional responses to negative stimuli (Yoo et al., 2007). Sleep deprivation also impairs the ability to accurately perceive emotional stimuli such as facial expressions (Van Der Helm et al., 2010; Killgore et al., 2017) and social cues (Dorrian et al., 2019), which can adversely affect the quality of interpersonal relationships and social support. Finally, clinical evidence suggests that sleep disorders contribute indirectly to suicide by virtue of their exacerbating effect on depression, a condition that in-and-of itself increases the likelihood of suicidal behavior (Britton et al., 2019). There is, however, growing evidence that sleep disruption can increase suicide risk exclusive of its effects on depression (Simmons et al., 2020). Overall, when sleep is disturbed or insufficient, positive mood state declines, and critical neurocognitive processes such as emotional awareness, decision-making, and self-regulatory capacities are degraded, which in concert may increase the propensity to contemplate, and perhaps, act on suicidal thoughts.

Despite the clear links between sleep and emotional functioning, the actual associations between sleep disturbance and suicidal thinking and behavior have also remained quite modest. This is not surprising, as considerable evidence suggests that there are stable trait-like interindividual differences in vulnerability and resilience to sleep loss (Van Dongen et al., 2004; Van Dongen and Belenky, 2009; Dennis et al., 2017; Yamazaki and Goel, 2020). In other words, some people appear to be inherently more susceptible to the adverse effects of insufficient sleep than others. This trait-like vulnerability appears to have some genetic basis (Goel et al., 2011; Casale and Goel, 2021) but has often been difficult to characterize through behavioral metrics. However, an early study by Taylor and colleagues showed that individuals high in the trait of extraversion tended to be more vulnerable to the effects of sleep deprivation on cognitive performance (Taylor and Mcfatter, 2003). Our team subsequently followed up on this work and further established the reliability of these findings, as extraverts were consistently more vulnerable than introverts to psychomotor vigilance decrements during two independent laboratory studies of total sleep deprivation (Killgore et al., 2007; Rupp et al., 2010). Consistent with the idea that extraverts are less resilient to mounting sleep pressure on alertness and vigilance, greater extraversion also appears to be associated with better sleep quality and fewer sleep problems in large population studies (Stephan et al., 2018). These findings are interesting and suggest that there is likely an underlying neurobiological factor associated with the dimension of introversion-extraversion that influences resilience to the effects of sleep loss on alertness and vigilance.

Early theories of personality postulated that extraverts may tend to have lower cortical arousal than introverts (Eysenck, 1967, 1981), a hypothesis that later received some support from neuroimaging studies (Johnson et al., 1999; Kumari et al., 2004). However, extraversion is also associated with higher impulsivity and lack of perseverance (Whiteside and Lynam, 2001), which could conceivably increase vulnerability to distraction and poor performance when sleep is lacking. As sleep deprivation is well known to lead to reductions in prefrontal cortex activity (Thomas et al., 2000, 2003; Wu et al., 2006) and reduced functional regulation of emotional responses (Yoo et al., 2007; Chee and Zhou, 2019), it would be reasonable to expect that individuals higher in extraversion would show more significant declines in cognitive control, attention, and vigilance during periods of sleep deprivation than those with more introverted traits.

Despite growing evidence that extraverts may be more vulnerable to mounting sleep pressure on attention and vigilance, this model has not been extended to potential changes in emotional outlook, such as suicidal ideation. Without sleep, it becomes difficult to regulate emotional responses and negative affect begins to hold greater sway over cognition (Walker and Van Der Helm, 2009). It would, therefore, not be unreasonable to expect that the previously identified vulnerabilities to vigilance decrements among extraverts during sleep deprivation would also extend to the emotional realm, and perhaps even impair the ability to inhibit potentially harmful thoughts/actions (e.g., suicidal ideation or self-harmful behavior) with such emotional dysregulation. Accordingly, we sought to determine the association between the personality trait of extraversion and vulnerability to increases in suicidal thinking during periods of insufficient or disturbed sleep. In a series of two studies, including a laboratory-based study of total sleep deprivation and a nationwide survey study of sleep and personality, we tested the hypothesis that greater trait-extraversion (TE) would be associated with greater suicidal ideation (SI) during total sleep deprivation (TSD) and among those with clinically significant insomnia in the general population.

## Study 1: Laboratory Sleep Deprivation

The goal of the first study was to directly examine the association between TE and SI over a controlled period of TSD. As part of a larger investigation on the effects of caffeine and alertness, military participants were recruited to complete an assessment of their personality, followed by three nights of in-laboratory TSD. Assessments of depression and suicidal ideation were collected at rested baseline and again following the second night without sleep. It was hypothesized that higher measured TE would be associated with greater increases in SI over the period of sleep deprivation, regardless of caffeine intake.

### Participants

Twenty-five U.S. Army enlisted personnel were recruited to participate in an extensive 5-day in-laboratory study. The sample included 20 males and five females, with a mean age of 25.4 years (SD = 4.1) and an average of 14.1 years (SD = 1.6) of formal education. Separate data from this study have been published previously (Killgore and Kamimori, 2020), but the present findings regarding the association between TE and suicidal ideation are novel and have not been previously reported. All volunteers underwent a physical examination to ensure they were healthy enough to remain awake for three days and potentially consume repeated doses of caffeine. Volunteers were excluded for any past or current physical/mental health/sleep problems, history of drug abuse, or current regular caffeine consumption ≥300 mg of caffeine per day, or a self-reported history of caffeine sensitivity, and all were healthy enough for currently active military service. Participants were also required to be on a normal day/night sleep schedule and report sleeping between 7 and 8 h per night, based on self-report. Current nicotine use was exclusionary and was verified by nicotine/cotine testing during the physical examination. Participants were required to abstain from alcohol, stimulants, and other psychoactive drugs for 48 h prior to the study. Written informed consent was obtained from all participants. This study was approved by the Walter Reed Army Institute of Research Human Use Review Committee and the U. S. Army Human Subjects Research Review Board.

### Materials

Participants completed several questionnaires at various points throughout the study. Trait Extraversion-Introversion (TE) was determined by the Extraversion Scale of the Revised NEO Personality Inventory (NEO-PI-R; Costa and Mccrae, 1992). The NEO-PI-R is one of the most widely used instruments for assessing the Big 5 personality traits including introversion-extraversion (De fruyt et al., 2000; Quirk et al., 2003; Sherry et al., 2003; Bagby et al., 2004). The NEO-PI-R includes five primary scales of Neuroticism, Extraversion, Openness to experience, Agreeableness, and Conscientiousness, which are conceptualized as independent and relatively orthogonal constructs that account for the majority of variance in personality. The scale provides normalized T-scores (i.e., normative Mean = 50, SD = 10) for each facet of personality, including trait Extraversion, or TE, which is the primary focus of the present study. The primary outcome variable, suicidal ideation, was assessed by the Suicidal Ideation (SUI) scale of the Personality Assessment Inventory (PAI; Morey, 2007). The PAI is a multidimensional assessment of various aspects of personality and psychopathological functioning. The inventory comprises 344 statements, each with four response options including: “False, not at all true,” “Slightly True,” “Mainly True,” and “Very True.” The SUI items focus on the presence of suicidal thoughts and the imminence of suicidal intentions. The items assess the tendency to think about suicide, the emotional desire to be dead or the belief that one would be better off dead, and the tendency to contemplate ways and means for killing oneself. The SUI scale is reported as a T-score based on comparisons to the normative group reported in the test manual. Higher scores indicate a greater severity and persistence of suicidal thinking. Additionally, because it is well established that depressive mood is associated with sleep problems, we elected to control for depressive mood in the analyses. To provide this control, we extracted the depression (DEP) scores from the PAI to be used as a covariate.

### Procedure

Each study session occurred over a 5-day period and participants were run in groups of 3–4 at a time. Participants reported to the lab at 19:00 h on Day 1 and underwent some familiarization and training with various tasks. From 23:00 (Day 1) to 07:00 (Day 2) participants retired to their own private bedrooms to undergo an 8-h baseline sleep opportunity. After awakening at 07:00 h (Day 2) participants remained awake for the next 77 h. At 15:05 on Day 2 (8 h awake), participants began the baseline PAI assessment, which lasted about 30-minutes. Then, at 16:15, they were administered the NEO-PI-R on a desktop computer to provide a baseline assessment of TE. After two days of TSD, at 15:05, participants completed a second administration of the PAI while in the sleep-deprived state (i.e., 56 h of continuous wakefulness).

Additionally, as part of a larger investigation into the effects of caffeine on psychomotor vigilance performance, each participant was randomly assigned to ingest either 200 mg of caffeine or placebo (four times during each overnight session—i.e., at 01:00, 03:00, 05:00, 07:00 h) in a chewing gum formulation (*Military Energy Gum^TM^*; MarketRight Inc., Plano, IL). While caffeine was expected to have been mostly cleared from the system by the time of the PAI assessment (i.e., the PAI was administered >8 h after the last dose of caffeine) and was not part of the study hypotheses, we nevertheless included caffeine group as a covariate in our analyses to assess and control potential influences of caffeine on outcomes.

### Analyses

Change scores from baseline were calculated for the SUI scale. Data were entered in a hierarchical multiple linear regression analysis to predict changes in PAI SUI T-scores using IBM SPSS 28. In the first step, caffeine group and changes in PAI DEP scores were forced into the equation. In the second step, all five primary factors from the NEO-PI-R (i.e., Neuroticism, Extraversion, Openness, Agreeableness, and Conscientiousness) were entered in a stepwise entry/deletion procedure (probability to enter = 0.05, probability to remove = 0.10). Finally, an interaction term was also entered to examine the interaction between extraversion and caffeine group.

### Results and discussion

**Figure 1:**
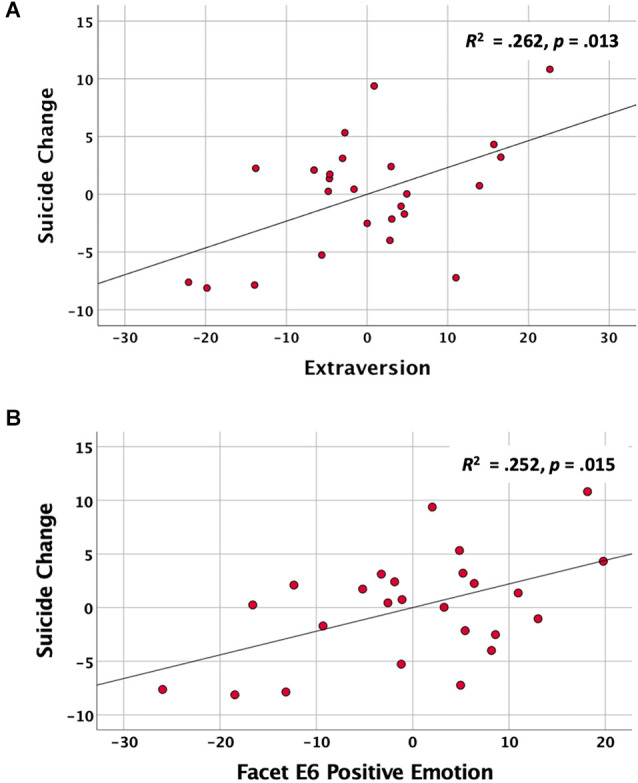
The associations between extraversion traits on the NEO-PI-R and change in suicidal ideation (SUI) scores over two nights of total sleep deprivation after controlling for caffeine intake and changes in depression. **(A)** Partial regression plot showing the association between baseline trait extraversion (TE) and change in SUI scores during sleep deprivation. **(B)** Partial regression plot showing the association between the Positive Emotion facet (E6) and changes in SUI scores during sleep deprivation.

All SUI scores remained within normal limits (i.e., T score <60) from baseline (min = 43, max = 54) to TSD (min = 43, max = 58), suggesting that any changes were within the sub-clinical range. In the first step, the caffeine group and changes in depression accounted for a non-significant proportion of variance in SUI change scores (*R*^2^ = 0.188, *p* = 0.102). However, at the second step, stepwise entry and deletion of the Big-5 personality traits resulted in only Extraversion being retained as a significant additional predictor of SUI change (*R*^2^ Change = 0.213, β = 0.463, partial *r* = 0.512, *p* = 0.013; see Figure 1A). After controlling for covariates, each 10-point T-score increment in TE translates into a 2.32-point T-score increase in SI from the rested to sleep-deprived state. Finally, when the caffeine group was included in the model as a categorical moderator, the interaction term was not significant, *p* = 0.693, and did not add any meaningful improvement in the model (*R*^2^ change = 0.005), suggesting that caffeine did not reliably affect the association between TE and changes in SI over the period of sleep loss. These findings support the primary hypothesis that individuals with higher TE tend to be more vulnerable to increased SI during a period of TSD, regardless of caffeine consumption.

It was also of interest to identify the facets of the extraversion scale that contributed most to the prediction of changes in SUI change. Accordingly, we calculated the partial correlations between SUI change and each of the six Extraversion Facet scores, after controlling for depression change and caffeine group. The Facets of Warmth (E1; partial *r* = 0.491, *p* = 0.017), Activity (E4; partial *r* = 0.436, *p* = 0.037), and Positive Emotions (E6; partial *r* = 0.502, *p* = 0.015) each separately correlated with change in suicide scores, while Gregariousness (E2; partial *r* = 0.271, *p* = 0.211), Assertiveness (E3; partial *r* = 0.233, *p* = 0.285), and Excitement Seeking (E5; partial *r* = 0.158, *p* = 0.472) did not. The heirarchical regression analysis with a stepwise entry of the six Extraversion Facets was used to identify facets that contributed unique variance to the prediction of SUI Change after controlling for changes in depression and caffeine group. This analysis showed that once the facet of Positive Emotions (E6, which measures the tendency to experience happiness, excitement, and joy) was entered into the equation (*R*^2^ change = 0.205, *p* = 0.015; Figure 1B), no other variables added uniquely to the prediction of SUI Change.

## Study 2: Large Scale Survey of Insomnia

While several nights of TSD within the context of a controlled laboratory setting can allow exceptional control and manipulation of sleep parameters, it is not clear whether similar effects would manifest in a more naturalistic setting, such as when sleep loss is due to chronic insomnia in a non-laboratory environment. Therefore, the goal of the second study was to extend the findings from Study 1, which focused on the association between extraversion and suicidal ideation in the context of TSD, to a more naturalistic setting to examine these associations in the context of self-reported sleep disturbance. Accordingly, in a large nationwide online survey, we collected data regarding insomnia severity, TE, and SI. Moreover, to further broaden the validity and generalizability of these associations, we incorporated alternative assessment metrics of TE and SI. It was hypothesized that higher measured TE would be associated with greater increases in SI among individuals meeting the criteria for clinically significant insomnia relative to those without such complaints.

### Participants

A total of 2,061 adults (43.8% male; 56.2% female) aged 18–79 (*M* = 36.8 years, SD = 12.2) were recruited *via* the Amazon Mechanical Turk (MTurk) online crowdsourcing platform (Litman et al., 2017) and were provided a small financial compensation for their participation. Participants were initially screened to ensure that they were geographically located within the United States (verified by IP address geo-coordinates), were at least 18 years of age, were able to read with at least a 6th grade proficiency and endorsed English as their primary language. No attempt was made to select individuals based on prior mental health, suicidal behavior, or other health-related factor. The racial/ethnic breakdown of the sample included 74.8% describing themselves as white, 10.6% as Black/African-American, 5.9% as Asian, 5.1% as Hispanic/Latino, 1.2% as Native American/American Indian/Native Alaskan, 0.2% as Native Hawaiian/Pacific Islander, and 2.2% responding as Other/Prefer not to answer. All participants provided electronic acknowledgment of informed consent after being provided with a full description of the study. This study was approved by the Institutional Review Board of the University of Arizona.

### Questionnaires and procedures

Participants were asked to complete several online questionnaires *via* the MTurk platform. Items were presented as worded in the original published versions of the questionnaires and responses were collected *via* button clicks in the online portal. There was no time limit imposed for completion, but most participants completed the survey in less than an hour.

#### Insomnia Severity Index (ISI)

While Study 1 focused on TSD, Study 2 focused on poor sleep due to insomnia. Accordingly, insufficient sleep was assessed with the Insomnia Severity Index (ISI), a well-validated and widely used index of insomnia, including difficulty falling asleep, staying asleep, and early morning awakening (Bastien et al., 2001; Morin et al., 2011). Higher scores on the ISI indicate greater severity of insomnia. For the present analysis, we categorized individuals as having insufficient sleep according to the commonly applied cut-off criterion of ≥10 as indicative of clinically significant insomnia (i.e., low or subthreshold sleep problems <10; high or clinical sleep problems ≥10). This cut-off has been found to have an optimal balance between sensitivity and specificity in population-based samples (Morin et al., 2011).

#### Eysenck Personality Inventory-Extraversion scale (EPI-E)

In Study 2, we chose to evaluate a different metric of TE than used in Study 1 to further extend the validity of the associations between the core constructs of extraversion, sleep loss, and suicidal ideation. Therefore, TE was assessed with the 24-item Extraversion Scale of the original Eysenck Personality Inventory (EPI; Eysenck and Eysenck, 1964). The Extraversion construct used in Eysenck’s scales has been extensively validated (Vingoe, 1968) and has a high correlation with the Extraversion scale of the NEO-PI (Draycott and Kline, 1995). Thus, the EPI provides an alternate but highly related metric of the construct of extraversion. Because of our focused hypothesis on extraversion, we only include the Extraversion Scale and not the other scales of the EPI. For the present analysis, EPI-E scores were calculated by summing the 24 items comprising the scale and were used as a continuous variable.

#### UCLA Loneliness Scale-3 (LS-3)

The present survey data were collected during the first year of the COVID-19 pandemic during a time when many individuals were required to maintain social distance and self-isolate. Our prior work has shown that many individuals struggled with loneliness during this period and that loneliness was significantly correlated with suicidal ideation during the pandemic (Killgore et al., 2020a, b). Therefore, we elected to also statistically control for the potential contribution of loneliness in the analyses. To provide this control, participants also completed the UCLA Loneliness Scale-3 (Russell, 1996), a well-validated measure of the construct of loneliness. The scale was scored according to standard instructions and was included as a continuous variable covariate in the regression analyses.

#### Beck Depression Inventory-2 (BDI-II)

Because insomnia is often highly correlated with depression, we wanted to statistically control for depressive mood state using the BDI-II (Beck et al., 1996). The full BDI-II includes 21 items that are each scored from zero to three for severity. The inventory is widely used for assessment of depressive mood and has been shown to have good to excellent psychometric properties (Wang and Gorenstein, 2013). However, since the BDI-II also includes a question about suicidal ideation, it was important to remove any variance associated with this suicide-specific item. Therefore, we calculated the total depression score without Item 9 of the BDI-II. Similarly, we also dropped Item 16 from the BDI-II since this item directly assesses changes in sleep patterns. The total score was the sum of the remaining 19 items, which provided a continuous variable assessing depressive mood, exclusive of suicidal thoughts and sleep problems.

#### Suicidal Ideation (SI) index

A composite suicidal ideation (SI) index was calculated by summing the total score for the suicidal ideation items from two commonly used screening indices for depression. First, Item 9 from the BDI-II was scored (range 0–3), with higher scores indicating greater severity of suicidal ideation. Second, we also administered the Patient Health Questionnaire (PHQ-9), which is a brief screener for depressive mood and also includes an item (Item 9) assessing the frequency of thoughts of suicide or self-harm. This item asks participants to indicate how often, over the past two weeks, they have been bothered by “thoughts that you would be better off dead or hurting yourself in some way.” It is important to note that the item can refer to either suicidal ideation OR other forms of self-harm, so it cannot be interpreted solely as a suicidal thinking item. The individual score for this item ranges from 0 (“not at all”) to 3 (“nearly every day”). The SI score was calculated by summing these two items from the two different depression scales, producing an index of suicidal or self-harming ideation that could potentially range from 0 (none) to 6 (high).

### Analyses

Data from the survey were analyzed using SPSS version 28. In a hierarchical multiple linear regression for categorical moderators (Aguinis, 2004), with PHQ-9 Suicide scores as the outcome variable, LS-3 and modified BDI-II scores were entered as covariates in the first step, followed by EPI scores and ISI category as the independent variables in the second step, and lastly, an interaction term representing the product of EPI-E score × ISI category was included. To ensure that the outcomes of the categorical analyses were not unduly influenced by the dichotomous nature of the cut-off values, the same analysis was also repeated with continuous values for the ISI. Additionally, to further elucidate the associations at each level of insomnia, a similar regression was calculated for the low and high ISI groups separately.

### Results and discussion

The SI index showed acceptable internal consistency for a simple 2-item scale, with Cronbach’s alpha = 0.74. Overall, most participants (64.4%) did not endorse any suicidal ideation on the SI index, while 35.6% indicated some evidence of thoughts about self-harm or suicide. Overall, 13.5% of the sample scored at least a 3 or higher on the 6-point scale.

**Figure 2:**
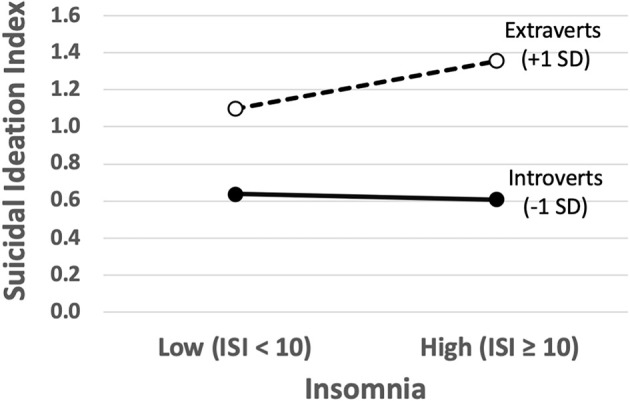
The plot of the interaction effect (moderation) between insomnia category (low or high) and trait extraversion from the Eysenck Personality Inventory (EPI) on suicidal ideation scores from the composite Suicide Ideation (SI) index. ISI, Insomnia Severity Index; SD, standard deviation.

Not surprisingly, at the first step of the regression analysis, the covariates (i.e., LS-3 loneliness and modified BDI-II depression) accounted for a significant proportion of the variance in SI scores (*R*^2^ = 0.411, *p* < 0.00001). Nonetheless, subsequent simultaneous entry of the primary predictor variables (EPI Extraversion and ISI Insomnia Category) was further associated with a significant improvement in the model (*R*^2^ Change = 0.039, *p* < 0.00001). Finally, the interaction term (i.e., Extraversion × ISI category) was highly significant (*R*^2^ Change = 0.009, *p* < 0.0001), suggesting that the association between extraversion and suicidal ideation differed between those with high and low levels of insomnia, as predicted, even after controlling for depression and loneliness. This interaction was plotted according to standard procedures (Aguinis, 2004) and is represented in Figure 2, which shows that for individuals with low extraversion scores (i.e., introverts), suicidal ideation was relatively low and did not differ as a function of insomnia severity, while high levels of extraversion tended to be associated with increased suicidal ideation, particularly for those with clinically elevated levels of insomnia. Table 1 provides the regression weights for the total model. For completeness in reporting, the model was again reanalyzed using ISI as a continuous variable, with nearly identical results (Table 2).

**Table 1:** Results of hierarchical linear regression predicting the combined suicidal ideation score from trait extraversion and *insomnia category*.

**R**	**R^2^**	**Variable**	**B**	**β**	**Test**	**Sig.**
0.678	0.459	Model			*F*_(5,2051)_ = 348.06	<0.001
		Constant	−1.180		*t*_(2051)_ = −9.797	<0.001
		LS-3	0.011	0.098	*t*_(2051)_ = 4.677	<0.001
		Mod. BDI-II	0.072	0.593	*t*_(2051)_ = 26.816	<0.001
		EPI Extraversion	0.054	0.168	*t*_(2051)_ = 9.007	<0.001
		ISI Category	−0.192	−0.070	*t*_(2051)_ = −3.352	<0.001
		Extraversion x ISI Category	0.034	0.122	*t*_(2051)_ = 5.839	<0.001

LS-3, UCLA Loneliness Scale-3; Mod. BDI-II, Modified BDI-II (i.e., without sleep item and suicide item included); EPI, Eysenck Personality Inventory; ISI, Insomnia Severity Index.

**Table 2:** Results of hierarchical linear regression predicting the combined suicidal ideation score from trait extraversion and *Continuous Insomnia Severity Index (ISI) scores*.

**R**	**R^2^**	**Variable**	**B**	**β**	**Test**	**Sig.**
0.676	0.457	Model			*F*_(5,2051)_ = 345.145	<0.001
		Constant	−0.879		*t*_(2051)_ = −5.969	<0.001
		LS-3	0.010	0.091	*t*_(2051)_ = 4.299	<0.001
		Mod. BDI-II	0.075	0.613	*t*_(2051)_ = 26.821	<0.001
		EPI Extraversion	0.021	0.066	*t*_(2051)_ = 2.029	<0.001
		ISI	−0.037	−0.183	*t*_(2051)_ = −4.620	<0.001
		Extraversion x ISI Score	0.004	0.240	*t*_(2051)_ = 5.185	<0.001

LS-3, UCLA Loneliness Scale-3; Mod. BDI-II, Modified BDI-II (i.e., without sleep item and suicide item included); EPI, Eysenck Personality Inventory; ISI, Insomnia Severity Index.

**Figure 3:**
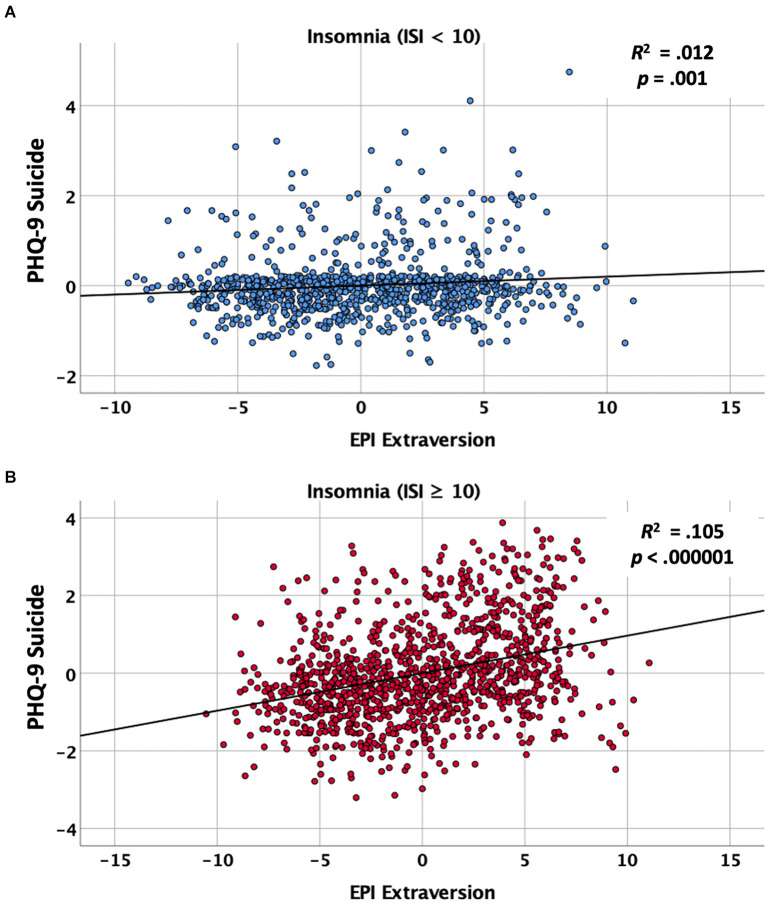
Partial regression plots showing the association between baseline trait extraversion as measured by the Eysenck Personality Inventory (EPI) and suicidal ideation on the composite Suicide Ideation (SI) index after controlling for loneliness and depression. **(A)** Partial regression plot for individuals scoring low (< 10) on the Insomnia Severity Index (ISI). **(B)** Partial regression plot for individuals scoring high (≥ 10) on the ISI.

Because the preceding analysis suggested that the associations between extraversion and suicidal ideation differed as a function of insomnia, we calculated the regressions again for the low and high insomnia groups separately. For those without clinically significant insomnia, the initial entry of the covariates (i.e., LS-3 loneliness and modified BDI-II depression) in the first step accounted for a significant proportion of the variance in SI index scores (*R*^2^ = 0.314, *p* < 0.00001). At the second step, EPI Extraversion was further associated with a modest but significant improvement in the model (*R*^2^ Change = 0.008, *p* = 0.001). As shown in the top panel of Figure 3, this analysis yielded a significant partial correlation between extraversion and suicidal ideation (partial *r* = 0.110, *p* = 0.001), suggesting that higher extraversion was modestly associated with greater suicidal ideation, even among individuals without sleep issues. Restricting the analysis to only those meeting the cut-off for clinically significant insomnia, we again found that the covariates accounted for significant variance in the SI index (*R*^2^ = 0.362, *p* < 0.00001). Moreover, the inclusion of EPI Extraversion significantly improved the model, accounting for more than twice the variance than the same model in the group without insomnia (*R*^2^ Change = 0.067, *p* < 0.00001). The bottom panel of Figure 3 shows that this analysis resulted in a significant partial correlation between extraversion and suicidal ideation (partial *r* = 0.324, *p* < 0.00001). Overall, these findings support the hypothesis that, among individuals with sleep disruption due to insomnia, greater levels of TE were associated with greater SI.

Finally, for completeness in reporting and to allow the full assessment of the robustness of the findings with and without covariates considered, we also ran an exploratory set of analyses that was identical to the preceding analyses, but without the covariates of depression and loneliness included. First, we ran the simpler model (i.e., without covariates) with the ISI as a categorical variable as before. Simultaneous entry of the primary predictor variables (EPI Extraversion and ISI Insomnia Category) was associated with a significant model (*R*^2^ = 0.118, *p* < 0.00001). Finally, the interaction term (i.e., Extraversion × ISI category) also added significant prediction (*R*^2^ Change = 0.046, *p* < 0.0001), suggesting that the association between extraversion and suicidal ideation differed between those with high and low levels of insomnia, as predicted, even without controlling for depression and loneliness. Similarly, the model was again reanalyzed using ISI as a continuous variable, with nearly identical results. Simultaneous entry of the primary predictor variables (EPI Extraversion and ISI Score) was associated with a significant model (*R*^2^ = 0.164, *p* < 0.00001). Finally, the interaction term (i.e., Extraversion x ISI category) also added significant prediction (*R*^2^ Change = 0.010, *p* < 0.0001). Overall, when the covariates were excluded the results remained similar to the primary analysis that included all covariates. These findings suggest that the results are robust regardless of the inclusion of the covariates of depression or loneliness.

## General Discussion

Insomnia, short sleep, and other sleep disorders are among several of the leading independent risk factors for suicide (Liu, 2004; Mccall and Black, 2013; Lin et al., 2018; Harris et al., 2020; Simmons et al., 2020). Nevertheless, suicide is a low base rate event relative to the prevalence of clinical sleep disturbance, which means that sleep problems (like most other risk factors) have low specificity and limited practical utility as warning signs for suicide (Harris et al., 2020). Consequently, it is vital to identify additional individual difference factors that may enhance the understanding and prediction of suicide risk in relation to sleep disruption. Here, across two independent studies, each with different data collection methods, assessment tools, and sleep outcome measures, we found converging evidence that the effects of sleep disturbance on suicidal ideation were significantly moderated by the individual’s level of introversion-extraversion, with higher levels of TE associated with a greater propensity for suicidal ideation when sleep was disturbed. Study 1 clearly showed that greater TE was associated with correspondingly larger increases in SI following two full nights of laboratory-controlled TSD. Individuals with the highest TE showed the greatest increases in SI from baseline to 56 h of sleep deprivation, whereas those with the lowest TE (i.e., more introverted individuals) tended to show the least increase in SI during sleep deprivation. Moreover, the effect was specific to TE (particularly the tendency to experience positive emotions such as excitement, happiness, and joy), as none of the other personality traits from the Big-5 added any additional predictive power to the model. Then, in Study 2, we extended these findings to a large nationwide sample focused on individuals classified according to self-reported presence or absence of clinically significant insomnia. In that study, we specifically focused on TE and found that the association between insomnia and SI was moderated by TE. Specifically, SI scores did not differ as a function of insomnia for people who were low in TE (i.e., more introverted), but SI differed significantly as a function of insomnia for high TE individuals. Together these findings suggest that there are significant inter-individual differences in how sleep deprivation/disturbance affects SI, which are moderated by a person’s level of TE. Low TE (i.e., more introverted) individuals appear to be modestly resilient to the effects of sleep disruption on SI, while those high in TE appear to be more vulnerable to increased SI during periods of sleep disruption. These individual difference findings parallel those reported for vigilance decrements during sleep deprivation, which also suggests that extraverted individuals tend to be more vulnerable to the effects of sleep loss on performance compared to more introverted individuals (Taylor and Mcfatter, 2003; Killgore et al., 2007; Rupp et al., 2010).

**Figure 4:**
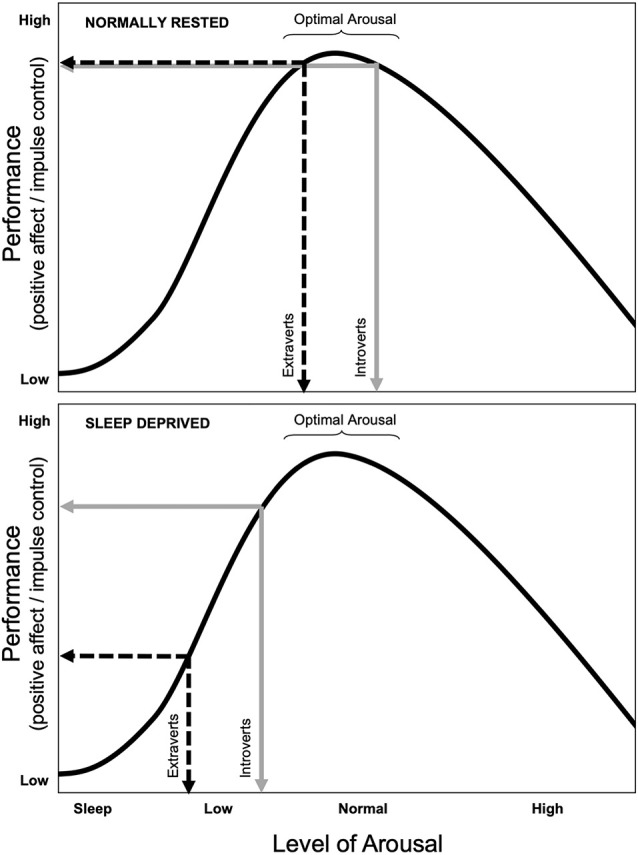
Diagram of the hypothesized effects of sleep loss (i.e., decreased arousal) on outcome measures (e.g., performance, positive affect, impulse control) based broadly on Eysenck’s theory of cortical arousal (1967, 1981). Top Panel: According to Eysenck’s theory, extraverts (dashed black line) function at a lower level of tonic cortical arousal than introverts (solid gray line); when normally rested, this would be expected to lead to similar levels of performance, positive affect, and impulse control. Bottom Panel: During sleep loss, arousal levels would be expected to decline for both groups equally (i.e., shifting both groups to the left), but would be manifested most noticeably among extraverts, as their tonic arousal exists closer to the left side of the curve than for introverts. This is postulated to lead individuals higher in trait extraversion to show greater mood declines and difficulties regulating suicidal impulses when sleep is lacking.

At present, the underlying mechanisms contributing to the moderating effect of TE on SI during periods of disturbed sleep have yet to be fully elucidated, but we discuss a few potential conceptualizations, none of which are necessarily mutually exclusive. One potential, but antiquated, explanation stems from the early conceptualization of TE, which proposed that the personality dimension of introversion-extraversion is a behavioral manifestation of individual differences in tonic cortical arousal (Eysenck, 1967, 1981). According to this early theory by Eysenck, individuals with greater TE tend to have lower levels of tonic arousal of the reticulo-thalamic-cortical activation system relative to more introverted (i.e., low TE) individuals. According to this early theory, the extraverted person is postulated to seek out social stimulation and excitement to modulate their cortical arousal to sustain an optimal level, while the introverted individual, whose baseline tonic arousal is inherently higher, avoids such stimulating activities to sustain their arousal within the optimal range. While the neurobiological basis of the theory is a bit obsolete, the general postulates of Eysenck’s theory have received support from a handful of neuroimaging studies, which have shown that introverted individuals tend to have greater basal cortical activation within expected brain regions compared to extraverts (Johnson et al., 1999; Kumari et al., 2004). Consistent with this notion, Taylor and McFatter found that individuals higher in extraversion were in fact more vulnerable to vigilance decrements during sleep loss (Taylor and Mcfatter, 2003), a finding that was replicated in two additional studies by our team as well (Killgore et al., 2007; Rupp et al., 2010). As shown in Figure 4, this general arousal model could be easily adapted to explain the effects of sleep loss on factors that could relate directly to suicidal ideation or behavior, including executive control, positive affect, or impulsivity. As suggested in the theoretical account outlined in the top panel of Figure 4, when normally rested, introverts and extroverts would be expected to function similarly when near their optimal level of arousal and would therefore not differ in terms of performance, positive affect, or impulse control. However, as shown in the bottom panel of Figure 4, in accord with the postulated lower level of tonic arousal among extraverts, a decline in arousal due to insufficient sleep might be expected to lead to an earlier and more noticeable decline in performance, positive affect, and/or impulse control than for introverts.

Regardless of the ultimate veracity of the cortical arousal theory of introversion-extraversion put forth by Eysenck, there is considerable evidence suggesting that extroverted individuals tend to be more impulsive than those with more introverted traits (Revelle, 1997). Of note, extraversion appears to correlate with one facet of impulsivity in particular. This facet of the construct is described as “a lack of perseverance,” or difficulty ignoring distracting stimuli to remain consistently focused on a specific task (Whiteside and Lynam, 2001). Since a decline in sustained and focused attention is one of the most reliably observed effects of sleep deprivation (Durmer and Dinges, 2005; Lim and Dinges, 2010), it is not surprising that extraverts would show greater vulnerability to this form of impulsivity during sleep loss (Taylor and Mcfatter, 2003; Killgore et al., 2007; Rupp et al., 2010). In a broader sense, impulsivity is a multidimensional construct that has also been used to describe a pattern of behavior characterized by a failure to inhibit inappropriate behavior and respond before considering the consequences of an action (Gvion et al., 2015). While most suicides have usually been contemplated for a while, impulsivity can increase the risk that an individual struggling with mental pain may decide to act on suicidal thoughts (Gvion et al., 2015). Thus, extraversion and associated tendencies toward impulsivity may further increase the vulnerability to suicidal decision processes when a distressed individual perceives no better alternatives.

Our proposition is that sleep disturbance may increase the vulnerability to suicidal decision processes, particularly among those higher in TE. This hypothesis emerges from the well-established evidence that insufficient sleep reduces metabolic activity within the prefrontal cortex (Thomas et al., 2000, 2003; Wu et al., 2006) and weakens functional connectivity among regions that regulate emotional reactivity (Yoo et al., 2007; Chee and Zhou, 2019). These alterations in normal brain functioning can have important consequences for suicidal thinking and behavior. First, the reduced prefrontal regulatory control of the amygdala produced by sleep disruption would be expected to increase the likelihood that life experiences may be viewed as more affectively salient and imbued with negative emotional tone than when normally rested (Walker and Van Der Helm, 2009). As negative emotions tend to increase during sleep deprivation (Killgore et al., 2008) or following poor quality sleep (Baglioni et al., 2010; Tkachenko et al., 2014), this lack of emotional regulatory capacity could exacerbate existing mental pain and thoughts of suicide for some individuals (Verrocchio et al., 2016). Second, consistent with the well-known role of the prefrontal cortex in higher order executive functioning and cognition, sleep deprivation has been shown to impair critical aspects of cognitive processing, including decision-making and risk propensity (Killgore et al., 2006; Dickinson et al., 2021), cognitive flexibility (Honn et al., 2019), as well as constructive thinking and emotional intelligence (Killgore et al., 2008). Deficits in many of these capacities have also been reported among individuals with chronic sleep disturbances (Cheng et al., 2017; Ballesio et al., 2019; Killgore et al., 2021). It is conceivable that, during a period of sleep disturbance, an individual may evaluate the perceived costs vs. benefits of suicide differently than when well-rested, leading to different decisions and behavioral outcomes. Third, lack of sleep has been shown to decrease metabolic activity in lateral prefrontal cortex regions involved in inhibitory capacity (Thomas et al., 2000; Muzur et al., 2002), which often translates to increased impulsive behavior (Demos et al., 2016; Saksvik-Lehouillier et al., 2020). In one study, sleep-deprived individuals demonstrated enhanced impulsive reactivity to negative stimuli (Anderson and Platten, 2011), which could conceivably amplify the tendency to act on suicidal thoughts, particularly among individuals with higher TE. Together, these findings suggest that insufficient or poor-quality sleep may contribute to functional brain changes that weaken regulatory control over negative emotions, degrade normal decision-making processes, and suppress inhibitory control of behavior—all of which do not portend well for someone in emotional pain and already contemplating suicide, particularly if they are already prone to impulsivity as a facet of high extraversion.

Of course, there are other potential explanations for these findings as well. Notably, we found that in Study 1, the association between TE and changes in suicidal thinking was primarily accounted for by differences in positive emotion (i.e., the tendency to experience high levels of happiness, excitement, and joy), rather than other facets. This raises the possibility that individuals who endorse such extremes in positive emotions may be particularly hard hit by the changes in prefrontal activation and accordant mood shifts produced by sleep loss, leading to greater feelings of hopelessness or despair under such circumstances. This possibility deserves additional investigation. It is also quite possible that individuals who are higher in TE and its accompanying positive emotions are more socially engaged during the day and that increased social interaction produces greater declines in cognitive resources. Social interactions require effort, including sustained self-monitoring and exertion of self-control, which can lead to a state sometimes referred to as “ego depletion,” in which the ability to self-regulate behavior becomes diminished (Baumeister, 2014). Ego depletion is known to be moderated by the trait of extraversion (Johnson et al., 2014) and appears to be exacerbated by sleep loss (Pilcher et al., 2015; Guarana et al., 2021). In one study, we demonstrated that social exposure led to greater vigilance decrements during sleep deprivation for extroverts compared to introverts (Rupp et al., 2010). Thus, it is possible that high TE individuals may simply be more vulnerable to a broad range of cognitive and affective declines when sleep deprived due to greater depletion of self-control resources produced by greater social engagement during the day compared to their low TE counterparts. We were not able to assess this possibility here, but the role of social effort and depletion of self-control among those higher in TE should be an area for further research.

While the aforementioned findings do not suggest that a person who is high in TE is destined to engage in suicidal behavior when sleep deprived, these findings do point to a potential contributing factor that may be important to incorporate into a more comprehensive understanding of suicidal phenomena. Consistent with the two-process model (Borbely, 1982), we speculate that during normal daytime hours, the gradual decline in self-control resources is likely offset by the upswing in circadian alertness, but as the period of wakefulness is extended beyond the normal sleep period, it likely becomes progressively more difficult to sustain a positive affective tone and suppress unwanted or unproductive thoughts. For the individual predisposed to a negative outlook, these thoughts may include considerations of self-harm or suicide. With further reductions of prefrontal cortical activation during sleep loss (Thomas et al., 2000) and the incumbent degradation of executive control over affect and behavior that emerges, the individual may find themselves experiencing an excessive negative emotion, self-critical thoughts, and impulsive tendencies. When combined with deficits in decision making, altered moral reasoning, and reduced inhibitory control, the risk for suicide can rise precipitously. Some evidence suggests that, after adjusting for the percent of the populace awake, suicides are most probable in the early morning hours, when sleep is lacking and the individual is functioning outside of their normal circadian phase (Perlis et al., 2016a, b; Tubbs et al., 2020). Moreover, being awake and alone at such times can further increase the risk of suicide as needed social support is absent (Calati et al., 2019), and there are few stimuli available to activate countervailing cognitive resources. Together, these factors can substantially increase the risk of suicide for the vulnerable individual. Our findings suggest that while neither being high in TE nor experiencing sleep disturbance is a sufficient clinical predictor of suicidal risk, when combined these factors do interact such that an individual who is high in TE may show a slightly elevated tendency to engage suicide-related cognitions. Of course, it is important to highlight that despite the significant interaction between extraversion and insomnia, the main effects of depression tend to explain the largest proportion of the variance, suggesting that careful assessment of depression remains the first line entry point for identifying suicidal thinking.

These findings should be interpreted with appropriate consideration of several limitations. For Study 1, which focused on multiple nights of sleep deprivation, the sample size was quite modest and included only military personnel, the majority of whom were male. Consequently, the generalizability of the findings will require additional replication in larger and more diverse samples. Additionally, the outcome measure was a self-report scale that included items related to suicidal cognition. Endorsing such items does not necessarily translate into suicidal behavior and all scores on the suicide scale were well within normal limits, suggesting that the current findings may not extend to clinically significant suicide. Nonetheless, the fact that significant changes were observed on such a scale in a tightly controlled environment attests to the potential robustness of the effect. Further, the study was extremely well controlled and participants were closely monitored, which allows confident conclusions that the associations were directly relevant to sleep deprivation. For Study 2, the findings are limited by the cross-sectional nature of the data collection, which makes it impossible to determine the directional causality of insomnia and suicidal ideation, although the findings are consistent with the outcomes expected from the experimental manipulation of sleep deprivation conducted in Study 1. Additionally, Study 2 also suffers from a self-report bias, as all metrics were collected online and *via* self-report instruments assessing suicidal ideation and insomnia. It is not possible to determine how these self-reported tendencies may translate into suicidal behavior. A strength of Study 2 is the large sample size, which allows greater confidence in the findings and considerable range in scores, with over 30% indicating that they had recently thought that they “would be better off dead.”

With due consideration to the limitations described above, we believe that the present series of studies adds to the understanding of suicide risk. While evidence suggests that suicide risk is greater when an individual is lacking sleep, when they are awake at a time when they should be sleeping, and when they are likely to be alone, our findings further suggest that individuals who are high in TE may be particularly vulnerable to these effects. The extent to which these factors are related to individual differences in brain functioning, arousal, social ego-depletion, or other mechanisms remains to be elucidated.

## Data Availability Statement

The raw data supporting the conclusions of this article will be made available by the authors, without undue reservation.

## Ethics Statement

The studies involving human participants were reviewed and approved by Walter Reed Army Institute of Research Office of Research Management; U.S. Army Human Subjects Research Protection Office; University of Arizona Institutional Review Board. The patients/participants provided their written informed consent to participate in this study.

## Author Contributions

WK: primary study design and conceptualization, primary literature search, data analysis, data interpretation, writing of the initial draft, figures, and tables. MG, AT, F-XF, TD, VC, and ND: contributed to study conceptualization, data interpretation, and editing drafts of manuscript. All authors contributed to the article and approved the submitted version.

## Funding

This work was was supported by U. S. Army Medical Research and Development Command.
